# Neurological Complications in Dengue Among Males of the Adult Age Group

**DOI:** 10.7759/cureus.51586

**Published:** 2024-01-03

**Authors:** Umesh Kakde, Mahalaqua Nazli Khatib

**Affiliations:** 1 Medicine, Jawaharlal Nehru Medical College, Datta Meghe Institute of Research and Higher Education, Wardha, IND; 2 School of Epidemiology and Public Health, Jawaharlal Nehru Medical College, Datta Meghe Institute of Medical Sciences, Wardha, IND

**Keywords:** neurological manifestations of denv, neurological complications, dengue virus, dengue shock syndrome, dengue haemorrhagic fever

## Abstract

Neurological problems are more frequently linked to dengue, a mosquito-transmitted virus common in tropical areas. This review study thoroughly examines the effects of dengue on adult males' neurological systems. Dengue hemorrhagic fever (DHF) or dengue shock syndrome (DSS) can develop in severe cases of dengue fever caused by the dengue virus (DENV). Unsettlingly, it is thought that a sizable portion of DENV infections impact the central nervous system (CNS), which calls into question the former theory that the DENV is not neurotropic. This review dissects the many neurological manifestations of dengue, spanning from encephalopathy, encephalitis, and other CNS implications to peripheral neuromuscular issues, through the systematic analysis of publications gathered from PubMed. The essay emphasizes the immunological reactions brought on by DENV infections and offers a deeper understanding of the pathophysiology. Given that they exhibit similar first symptoms, Zika and chikungunya are two more illnesses that must be distinguished from dengue. The mainstay of current diagnostic methods is serum and cerebrospinal fluid (CSF) tests, although supportive care is still used. This review highlights the importance of tracking neurological symptoms in dengue patients and encourages more studies in this area.

## Introduction and background

The dengue virus (DENV) is the source of dengue fever, a mosquito-transmitted tropical disease. Typically, three to 14 days after the infection, symptoms appear. These symptoms can include a high body temperature, headache, nausea, muscle and joint discomfort, and the typical rash and itching of the skin. Usually, it takes two to seven days to recover. The illness progresses to a more severe form of dengue hemorrhagic fever (DHF) that causes bleeding, low blood platelet counts, and plasma leakage, or to dengue shock syndrome (DSS), which causes dangerously low blood pressure. The second most prevalent illness spread by mosquitoes to humans is dengue [1]. In 2009, the WHO approved new recommendations that, for the first time, classify severe dengue cases according to their clinical condition, which includes neurological signs. Depending on the clinical setting, dengue has been associated with various neurological symptoms in 0-21% of hospital patients [2-3]. A single positive-stranded RNA virus belonging to the family *Flaviviridae* and genus *Flavivirus*, dengue is spread via mosquitoes. The *Aedes aegypti *mosquito is the vector. In India, it is a widespread endemic viral infection. Due to heavy rains, inadequate sanitation, stagnant water, and ineffective mosquito control, dengue is a significant public health concern in India. Thirty-four out of the 96 million dengue cases worldwide in 2010 were located in India. In a study from Pune City, the seroprevalence of dengue infection in patients with fever was 21.65% [4].

A flavivirus called the DENV significantly contributes to human illness, with annual medical and mosquito control expenses in billions of dollars [5]. Up to 20% of DENV infections are thought to impact the brain [6]. DENV infections are becoming increasingly common, so more people may be at risk of experiencing neurological problems. The multisystem dysfunction brought on by the DENV, which resulted in encephalopathy, has been linked to central nervous system (CNS) problems. It was first believed that the DENV was not a neurotropic virus and neuro-invasion is being demonstrated by immune-histochemistry identification of the DENV antigen in the brain in people with fatal dengue encephalopathy and also by polymerase chain reaction (PCR) and IgM antibody tests in cerebrospinal fluid (CSF) for patients with dengue encephalitis [7]. Only a few case studies have discussed the severe neurologic consequences of dengue fever and stroke. Uncertainty exists on the prevalence and risk factors for stroke in dengue patients.

## Review

Search methodology

Resources from the World Health Organization (WHO) and Centers for Disease Control and Prevention (CDC) were also used, along with electronic databases like PubMed, Google Scholar, and Scopus, to compile the data for the dengue fever article. Books and periodicals important to dengue and related illnesses were included in the literature. Keywords and Medical Subject Headings (MeSH) concepts, such as "dengue fever," "epidemiology of dengue," and "pathogenesis," were incorporated in search queries. Boolean logic increased the accuracy of searches. The selection criteria were stringent. Only recent research with information on worldwide epidemiology, the biology and transmission of the DENV, or the pathogenesis, complications, and therapy of dengue were considered. The following were excluded: non-English articles, studies older than 10 years (unless seminal), and studies with unclear techniques or unreliable data.

**Figure 1:**
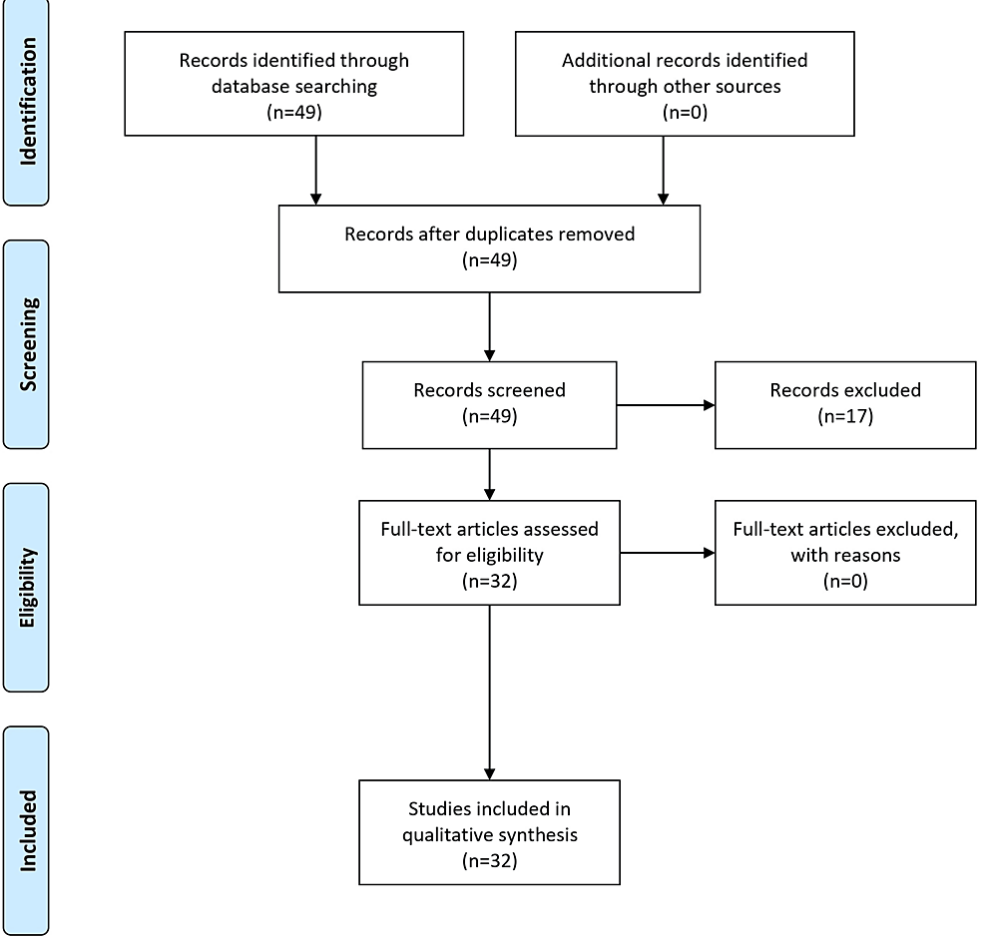
PRISMA flow diagram PRISMA: Preferred Reporting Items for Systematic Reviews and Meta-Analyses

The full-text publications were carefully evaluated after ensuring relevance by analyzing the titles and abstracts. Additional information was gathered from the references of chosen articles. Prevalence rates, clinical symptoms, and treatment strategies were the main areas of emphasis throughout data extraction. Critical analysis revealed common themes and inconsistencies combined to create a coherent article. The Newcastle-Ottawa scale and the Cochrane Collaboration tool were used in quality checks to ensure the article's integrity. Because research on infectious diseases is constantly changing, reviewing and updating the search strategy every two to three years is advised to ensure that the information is still relevant. The Preferred Reporting Items for Systematic Reviews and Meta-Analyses (PRISMA) flow diagram is shown in Figure 1.

Epidemiology

The global prevalence of dengue fever has recently increased, placing nearly half of the world's population in danger. Approximately 100-400 million infections occur yearly, with more than 80% exhibiting mild or no symptoms. As a result, many dengue cases go unreported or are misdiagnosed [1]. A model forecasts 390 million annual dengue infections (with a 95% confidence interval (CI) of 284-528 million), with 96 million (varying from 67 million to 136 million) displaying visible clinical signs [2]. Even though there is a threat in 129 countries, Asia bears 70% of the load [3]. Dengue cases have increased more than eightfold in the last two decades, from 505,430 in 2000 to over 5.2 million in 2019 [4]. Dengue deaths increased from 960 in 2000 to 4032 in 2015, primarily affecting young people [4].

Although there appeared to be a decrease in cases and deaths in 2020 and 2021, the data are not definitive, and the COVID-19 situation may have influenced reporting in many places. Population growth, unchecked urban development in tropical and subtropical areas with unclean conditions, inconsistent infrastructure for water, ineffective garbage handling, and the rise of non-biodegradable containers all contribute to dengue fever's global spread. The absence of efficient methods to control mosquitoes and increased individual movement exacerbate the situation [5]. DENV evolution's quick progress into additional aggressive strains to hasten disease's spread. Around 500,000 individuals, primarily children, are hospitalized yearly due to dengue. Approximately 2.5% of these people die from the condition, reaching an estimated 25,000 global deaths annually. Significant outbreaks have resulted from changes in the main DENV serotypes. Dengue and transmission are shown in Table 1.

**Table 1:** Dengue and transmission Source reference: PubMed [6-8]

Category	Details
Virus family	Flaviviridae
Genus	Flavivirus
Structural proteins	Core, membrane, envelope
Serotypes	DENV-1 to DENV-4
Non-structural proteins	7 (NS)
Regions of coexistence	Tropical and subtropical areas
Main reservoir	Humans
Transmission	Mosquitoes (after biting an infected person)
Extrinsic incubation period	Duration between a mosquito ingesting infected blood and becoming a carrier. Temperature-dependent.
Mosquito lifespan	30-45 days
Notable mosquito species	*Aedes aegypti* (originated in Africa), *Aedes polynesiensis* (South Pacific), *Aedes scutellaris* (South Pacific), *Aedes albopictus* (originated in Southeast Asia)
*Aedes albopictus* traits	Adaptable to urban and suburban areas, migrated due to trade in worn tires, survives in colder weather, broad diet
Regions affected by *Aedes albopictus*	Cooler regions, including some Eastern European and Mediterranean nations

DENV and vectors

DENVs are single-stranded RNA viruses from the Flaviviridae family and the *Flavivirus* genus. Three identified structural protein genes, namely, core, membrane, and envelope (DENV-1 to DENV-4), and seven non-structural (NS) protein genes make up its RNA genome [6]. Within these serotypes, several genotypes with various levels of pathogenicity occur. All four DENV serotypes can coexist in tropical and subtropical areas and are common in urban and periurban environments. Although nonhuman primates can catch the virus in some parts of West Africa and Southeast Asia, humans are the DENV's main reservoir. After a mosquito bites an infected person, the virus multiplies and spreads to other people when humans are in the viremic phase. The extrinsic incubation period is the time between a mosquito ingesting infected blood and becoming a carrier. Being temperature-dependent, the mosquito cannot spread the virus through a bite until this period has passed. An infected mosquito can even spread the virus to its offspring and remain that way for its regular 30-45-day lifespan [7]. *Aedes aegypti *started in Africa and expanded worldwide because of trade, but in the South Pacific, *Aedes polynesiensis *and *Aedes scutellaris* are frequently encountered. Native to Southeast Asian woodlands, *Aedes albopictus* has adjusted to both suburban and urban settings [7]. This species recently migrated across continents, starting with Asia, partly due to the exchange of old tires. They can go for several months without water, but their eggs cannot. It can survive away from populated areas, thanks to its capacity to adapt to colder weather and broad diet, which includes food from various animals and birds. *Aedes albopictus* is becoming a big issue for dengue transmission in cooler regions, such as some Eastern European and Mediterranean nations [8].

Pathogenesis

As previously stated, any of the four serotypes (DENV1 through DENV4) can cause dengue. Any of these serotype infections can result in dengue fever. Such an infection provides lifelong immunity against identical subtype yet unsuitable for infections caused by different serotypes. The likelihood of developing hemorrhagic fever caused by dengue with later viral infections with different serotypes is, interestingly, increased if a history of prior infection with a different serotype exists [9]. Antibody-dependent enhancement (ADE) is the name for this intriguing and seemingly contradictory occurrence [10]. In ADE, several non-neutralizing antibodies combine with the DENV to increase the virus' ability to infect mononuclear phagocytes. As a result, host cells become more infected, virus replication is accelerated, and clinical symptoms worsen. There may be a connection between this process and the development of neurological and other disorders. The current SARS-CoV-2 epidemic could potentially give rise to a similar problem. Endothelial dysfunction that results in hemorrhage in the vessels and sign of serious dengue fever is elevated permeability. Despite the potential for significant repercussions, this frequently reverses wholly and quickly, suggesting the participation of inflammatory mediators as opposed to an actual endothelium infection [11].

The potential culprits include inflammatory molecules, such as tumor necrosis factor-a (TNF-a), frequently increasing throughout crucial dengue episodes. A soluble viral protein called dengue NS1 can similarly damage the endothelium glycocalyx and cause vascular leakage. The discrepancy between the commencement of vascular leakage and the time of NS1 antigenemia refutes this notion. Leukotrienes, platelet-activating factor (PAF), and other inflammatory lipid mediators also increase during acute dengue stages. In addition, the levels of vascular endothelial growth factor and angiopoietin-2 are raised in DHF patients, stimulating the phospholipases necessary for PAF synthesis. Interleukin-1b and inflammatory cytokines, whose platelets produce via monocytes, may decrease endothelial function. Therefore, treatments for severe dengue forms that target the downstream immune cascade may be helpful. The authenticity of the endothelium glycocalyx layer (EGL) also appears to be structurally compromised [12]. EGL breakdown has been linked to increased leukocyte adhesion with the endothelium, coagulation problems, and fluid extravasation, all linked to increased plasma leakage, although the precise process is yet unknown.

Neurological complications

The revised classification of dengue-related neurological symptoms aims to distinguish between dengue-related effects on the CNS and eyes, the PNS, and dengue-related immune-related disorders that develop later. CNS symptoms can appear in dengue fever, hemorrhagic fever, and DSS. According to the WHO's guidelines, a verified diagnosis of DENV infection is necessary to determine whether the virus causes a neurological disease. This can be determined by any of the following signs: a positive PCR, a positive virus culture, a change in the levels of immunoglobulin M (IgM) in successive serum samples, or a fourfold increase in the levels of IgG in consecutive serum samples [13]. Any of the following symptoms, such as altered awareness levels (with a Blantyre coma score less than 4 for children under five and a Glasgow coma score less than 14 for those over five), neck rigidity, particular neurological symptoms, or convulsions, can be used to diagnose dengue's impact on the CNS1. Dengue's neurological symptoms are shown in Table 2.

**Table 2:** Dengue's neurological symptoms Source reference: PubMed [13]

Category	Details/criteria
Revised classification aims	1. Effects on the CNS and eyes, 2. effects on the PNS, 3. dengue-related immune-related disorders
Conditions with CNS symptoms	1. Dengue fever, 2. dengue hemorrhagic fever, 3. dengue shock syndrome
Diagnostic requirement (according to the WHO)	Verified diagnosis of DENV infection
Signs for DENV infection	1. Positive PCR, 2. positive virus culture, 3. change in the levels of IgM in successive serum samples, 4. fourfold increase in levels of IgG in consecutive serum samples
Symptoms to diagnose dengue's impact on the CNS	1. Altered awareness levels with a Blantyre coma score less than 4 (for children under 5), 2. altered awareness with a Glasgow coma score less than 14 (for those over 5), 3. neck rigidity, 4. specific neurological symptoms, 5. convulsions

Dengue encephalopathy

The neurological condition most frequently linked to DENV infection is encephalopathy. The causes of dengue encephalopathy can originate from the more considerable illness. Issues including anoxia, brain swelling, low salt levels, protracted shock and internal haemorrhage, abrupt liver and kidney failure, and introduction of poisonous drugs may bring them on. DHF/DSS was previously believed to be the only factor contributing to dengue encephalopathy. By contrast, just 0.5% of the 5,400 patients with confirmed dengue diagnosed with DHF in a two-year study conducted in Vietnam displayed symptoms of dengue-associated encephalopathy [14]. These patients' EEGs might show patterns, such as burst suppression, seizures, or *Epilepsia partialis* continuous [15]. In most dengue encephalopathy cases, the CSF profile is unaltered, and brain scans may either show no abnormalities or broad cerebral oedema [16]. According to recent research, a DENV infection damages endothelium cells by producing too many cytokines, resulting in various CNS symptoms. Enzymes and cytokines, such as IL-1, TNF, and MMP2, play a crucial role in endothelial degradation, which increases vascular permeability and fluid leakage [12]. The swelling of the brain can result from this. Depending on the underlying causes and the standard of medical care, different patients with dengue encephalopathy experience different treatment outcomes. Lack of supportive care can increase the mortality rate. In one study from Sri Lanka, of 15 dengue encephalopathy patients (all diagnosed with DSS), nearly half passed away from the illness. In these instances, acute liver failure, electrolyte abnormalities, and shock were the leading causes of encephalopathy [17].

Dengue encephalitis

We now have a thorough grasp of the brain damage brought on by the DENV's neurotropic effects and direct CNS invasion over the past 10 years. Patients with dengue encephalitis may have symptoms, such as altered behavior, fever, headaches, nausea, vomiting, seizures, and diminished consciousness [18]. Interestingly, more than half of these patients with encephalitis do not exhibit the classic signs of dengue fever, such as rashes, muscle discomfort, or bleeding. The DENV-associated encephalitic condition can be accurately diagnosed using PCR and immunological assays on serum and CSF [19]. Seizures, particular neurological symptoms, serum and CSF samples with positive dengue PCR results, NS1 antigen, or detectable IgM dengue antibodies and the disregard for other possible viral encephalitis reasons are all indications of dengue encephalitis. Specific symptoms, including spontaneous microhemorrhages, are frequently seen during head CT scans. The thalamus, basal ganglia, and other damaged brain regions can all be precisely located with an MRI. Notably, different MRI sequences may reveal distinct features in particular brain regions. Differentiation is essential because diseases like ADEM, Japanese B encephalitis, and chikungunya can all cause comparable lesions. Getting a firm diagnosis of DENV involvement in the CNS can be challenging. The sensitivity of these tests is frequently inadequate, even though finding particular dengue markers in the CSF can diagnose dengue encephalitis. Therefore, a diagnosis often depends on clinical suspicion, confirmation of systemic DENV infection, emergence of encephalitic symptoms, probable CSF abnormalities, and particular findings in brain imaging.

Visual coherence tomography (OCT) and infrared fundus photography developments have increased the frequency with which visual disorders associated with dengue are now recognized and investigated. Ocular symptoms are frequently reported, ranging from mild problems like maculopathy and blurred vision to more serious ones like retinal detachment and oculomotor nerve palsy. Visual abnormalities, eye pain, redness, and photophobia are some symptoms that typically point to eye involvement. Ocular involvement in dengue infections is underreported because some lesions, particularly those in the peripheral retina, may not be symptomatic and, hence, be challenging to find. Partially unknown are the particular mechanisms underlying these eye problems. Low platelet counts may cause hemorrhagic symptoms, but other mechanisms, including inflammation and immunological reactions, have also been proposed. Intra-retinal haemorrhages, which can have a variety of appearances, are the hallmarks of specific visual symptoms, including dengue-related maculopathy. OCT can detect the lesion at the fovea, which is yellow-orange that characterizes dengue-related foveolitis. OCT can be used to distinguish between different forms of macular oedema, which is a typical symptom of maculopathy linked to dengue. A common sign of dengue-related optic neuropathy, whose underlying pathophysiology is still not fully understood, is inflammation and oedema of the optic disc [16]. Several variables, including stress from the viral infection and genetic influences, may contribute to central serous chorioretinopathy, which is still unknown in dengue. Steroids may be helpful in situations where an autoimmune reaction is thought to be at play, even though many dengue-related ophthalmic disorders usually get well on their own. They are not advised, though, when the virus is acutely present.

Immune‑mediated neurological syndromes

Mononeuropathies

Following an about of dengue fever, cranial nerve difficulties might develop, including ocular neuritis, problems with the oculomotor nerve, delayed thoracic neuropathy, isolated phrenic nerve palsy, and isolated sixth nerve palsy, especially Bell's palsy [20]. Usually, a diagnosis is made by ruling out alternate diagnoses. These problems' most likely root cause appears to be immunological responses. Supportive care is the primary therapeutic strategy, while early corticosteroid injection may be advantageous. DENV infection has been associated with polyradiculoneuropathies, lumbosacral plexopathies, Guillain-Barré syndrome (GBS), and its variations [21]. GBS symptoms might appear immediately or later in the course of the illness. Although the precise cause is still unknown, it is broadly accepted that it results from an immunological reaction. This is due to the possibility that dengue immunoglobulins could interact with peripheral neurons that have similar response patterns. The myelin sheath or the axons may be the target of such an immune response, resulting in demyelinating or axonal types of polyneuropathy [21].

Acute Transverse Myelitis

Acute transverse myelitis associated with dengue is extremely rare. It may become apparent before or after the infection. Usually, a large portion of the spinal cord is affected. In the post-infectious phase, it is thought that the immune system is to blame, while in the para-infectious stage, it is thought that a direct viral attack is to blame [22]. The optic nerve has not been mentioned in any known cases up to this point. The emergence of myelitis caused by a post-infectious immune response is typically one to two weeks following the start of the first symptoms. By contrast, The first week after infection is when para-infectious myelitis may manifest. On MR imaging, diagnostic procedures show alterations in the spinal cord's signal and edema. The CSF contains indications of dengue-specific IgG antibodies, and the virus RNA can also be extracted [22]. However, it is crucial to exclude diseases, such as neuromyelitis optica spectrum disorder (NMOSD) or multiple sclerosis (MS) with pertinent diagnostic testing, even if there is a clear link to a recent dengue infection.

Acute Disseminated Encephalomyelitis (ADEM)

After a DENV infection or DHF, acute disseminated encephalomyelitis (ADEM) may manifest itself throughout the healing process [23]. Seizures, shifts in awareness, and some neurological issues are common early symptoms that often appear after the feverish period. The CSF may show a modest rise in protein levels and a minor increase in cell count. T2-weighted and fluid-attenuated inversion recovery (FLAIR) imaging of the brain's white matter, particularly in regions like the centrum semiovale, corona radiata, and others, frequently show abnormalities. The thoracic and cervical regions of the spinal cord are most frequently affected by changes in spinal cord signals [23]. These lesions' perivascular lymphocyte presence with hemorrhagic regions, an influx of macrophages, and perivenous demyelination have all been highlighted by histological analyses.

Neuromuscular complications

Dengue‑Associated Hypokalemic Paralysis

Hypokalemic paralysis may be seen in patients who experience a sudden onset of limb weakness without impacting their cranial nerves, bladder, or bowel functions. Finding low potassium levels in the blood is necessary to confirm this disease. Nevertheless, it is crucial to monitor serum potassium levels and test urine for porphobilinogen in all patients suspected of having GBS, if it is connected to dengue. Paralysis caused by hypokalemia is often indicated by a blood potassium level of 3 mmol/liters or less. A weakness typically appears between the second and fifth day of the fever and worsens within four to 24 hours [24]. Patients frequently show impaired or nonexistent muscular responses. It is unclear what causes explicitly low potassium levels during dengue illness. A few suggested mechanisms are as follows [24]: The potential metabolic alkalosis brought on by excessive IV fluid consumption, especially lactate-containing IV fluids, might cause a potassium shift internally. Because of the systemic consequences of the infection, potassium is redistributed between cells and extracellular fluid. Potential kidney issues that could cause excessive potassium loss in the urine. Stress-related hormones are released, pushing potassium into cells and resulting in a fall in serum potassium. Specific genetic mutations in the calcium and sodium channel genes have been connected to some cases of hypokalemic periodic paralysis. Hypokalemic paralysis linked to dengue may result from a similar channel dysfunction that the virus either causes or makes apparent.

Rhabdomyolysis

The damage to muscle cells brought on by the release of cytokines, particularly TNF and interferon alpha, which are sparked by the DENV, is thought to be the cause of dengue-related rhabdomyolysis. Increased cytokine levels increase the quantity of free calcium in cells, either due to decreased adenosine triphosphate (ATP) levels or because the cell membrane is directly harmed. This increase in intracellular calcium activates proteases, causes mitochondrial problems, and produces excessive reactive oxygen species, which can harm muscle cells. Muscle cells eventually perish as a result of these processes [25]. Rhabdomyolysis can also cause severe renal problems (AKI) and serious electrolyte abnormalities.

Myalgias

During the initial stages of the illness, the typical signs are muscle pain, sensitivity, and slight swelling. This pain predominantly impacts the back and muscles near the limbs, leading to walking challenges without any discernible weakness. The pain is potentially due to the virus directly attacking the muscles, followed by subsequent inflammatory responses. Although electromyography (EMG) is typically average [26], minor myopathic changes are detected in cases where creatinine kinase levels are elevated. When examining the tissue, one might notice moderate infiltration of mononuclear cells around blood vessels, lipid buildup, minimal growth of mitochondria, occasional centralized nuclei, instances of muscle cell death, and muscle fiber clustering [26]. Generally, muscle pain tends to be brief and resolves on its own.

Cerebellar Syndromes in Dengue

Possible causes of DENV-related cerebellar disorders include a minor immune-driven inflammatory response. They may appear at the height of the infection or one to three weeks after the severe symptoms of dengue have passed [27]. These cerebellar disorders typically seem to improve independently [27]. It is important to rule out viral infections even if they can cause similar symptoms. ADEM is another primary condition to distinguish from. The accuracy of clinical diagnosis is still high in regions with a high prevalence of DENV infection, particularly in patients that fully match the requirements for DHF [28]. Fever, bleeding manifestations (proven by a cutaneous or mucosal bleeding, such as gastrointestinal bleeding, and a positive tourniquet test, nosebleeds, or heavy menstrual bleeding), low platelet count (100,000 cells/mm^3^), and evidence of plasma leakage (e.g., pleural effusion, ascites, significant hemoconcentration, or low protein levels) are the four specific criteria that must be met to diagnose DHF [28]. DSS is characterized by circulatory collapse and DHF [28]. Dengue, chikungunya, and Zika can all have very similar first symptoms. Although a laboratory test to confirm the diagnosis is preferable, the findings sometimes do not come in time to guide the initial therapy choices.

Laboratory Testing

DENV infection can be diagnosed directly by looking for viral components in serum or indirectly through serology. The timing of sample collection about the onset of the patient's symptoms will determine how accurate each approach will be. Despite being more precise, antigen serology is more cost-effective but less accurate than PCR for detecting the virus's nucleic acid. Reverse-transcriptase PCR or the viral antigen NS1 (often positive in the first seven days) can detect the virus' nucleic acid in serum during the first week of the sickness. Over 90% of initial infections may be detected with NS1, and the antigen stays present long after the fever has subsided. However, in secondary infections, its sensitivity falls to 60-80% [29]. It is possible to detect IgM as soon as the fourth day of symptoms. Its discovery in serum samples from individuals exhibiting symptoms is a typical method of confirming dengue fever [30]. A considerable rise in antibody levels between the initial and later samples provides additional proof. Whether an infection is primary or secondary affects the likelihood of finding IgG. While secondary infections have a rapid, high-titered, cross-reactive response, primary infections exhibit a slow, low antibody response [30]. It can be challenging to diagnose dengue in those who recently received vaccinations, exposure to the virus, or vaccinations against Zika or yellow fever. Another way is to isolate the DENV (via culture), but because it takes so long, it is rarely employed in clinical settings. Tissue samples can detect dengue viral proteins, with the liver offering the best chances [31]. However, in cases when the DENV is suspected, biopsies are rarely necessary.

Treatment

There is not a specific treatment for dengue fever as of yet. Treatment focuses on supportive care, which includes controlling temperature, continuously monitoring blood pressure, giving fluids, and, if required, transfusing blood or platelets. Aspirin should not be administered to children to reduce the danger of Reye's syndrome. It is crucial to manage fluids properly. Hematocrit levels that significantly rise signify severe fluid loss and demand rapid fluid replacement. For this purpose, crystalloids are advised by the WHO1; for patients who do not respond to crystalloids, colloids and blood can be considered. In addition to the usual maintenance fluids based on weight [13], it is recommended to administer a fluid volume equivalent to 10 ml/kg for every 1% loss in body weight. If there has been significant bleeding, platelet transfusions may be required. It is essential to properly manage any complications, including electrolyte imbalances, liver or kidney problems, or low blood sugar [32]. Brain-related symptoms have no specific treatment, and neither corticosteroids nor antiviral medications have demonstrated clear advantages in an ICU. Medications like anti-seizure medications or substances to lessen brain swelling can be required. Treatments such as high-dose corticosteroids or IV immune therapy can help conditions brought on by immunological reactions. Being immune to one serotype of DENV only provides temporary protection and does not protect against the other serotypes, which is a regrettable aspect of DENV infection.

## Conclusions

The viral virus dengue, which is spread by mosquitoes, is becoming a serious global public health issue. Its incidence has increased globally, affecting large populations and taxing healthcare systems, particularly in Asia. This in-depth analysis examined the many features of dengue, including its epidemiology, the complex interactions between its virus and vectors, its pathophysiology, and the wide range of neurological disorders brought on by the infection. Even though the majority of dengue infections are mild or asymptomatic, the possibility of serious symptoms, such as DHF, DSS, and other neurological sequelae, calls for more awareness and caution in clinical treatment. Our improved understanding of the neurological symptoms of dengue, which include encephalopathy, encephalitis, and a variety of neuromuscular problems, highlights the intricacy of the virus and its wide-ranging effects on the human body. Clinicians must stay up to date on these manifestations in order to make an early diagnosis and provide the proper care.

Although there have been improvements in diagnostic approaches, difficulties still exist. The need for ongoing improvement in diagnostic techniques is highlighted by the clinical symptoms that overlap with those of other illnesses, especially Zika and chikungunya, as well as the limitations in diagnostic sensitivity, particularly in secondary infections. Last but not least, the dengue treatment landscape continues to be mainly positive. Although specific antiviral therapies are difficult to come by, managing patients effectively requires an awareness of the course and complications of the illness. This assessment serves as a necessary reminder of the complexity of dengue and the need to fund both research and public health initiatives to lessen its impact on the world, given the ever-changing landscape of infectious illnesses.
